# Longitudinal Assessment of Fatigue in Pregnancy Complicated by Cervical Cancer: A Prospective Case Study and Implications for Nursing and Midwifery Practice

**DOI:** 10.3390/nursrep15030108

**Published:** 2025-03-19

**Authors:** Anna Weronika Szablewska, Agata Zdun-Ryżewska

**Affiliations:** 1 Division of Obstetric and Gynaecological Nursing, Faculty of Health Sciences with the Institute of Maritime and Tropical Medicine, Medical University of Gdansk, 80-210 Gdansk, Poland; 2 Division of Quality of Life Research, Department of Psychology, Faculty of Health Sciences with the Institute of Maritime and Tropical Medicine, Medical University of Gdansk, 80-210 Gdansk, Poland; azdun@gumed.edu.pl

**Keywords:** cancer in pregnancy, cervical cancer, fatigue, case study

## Abstract

**Background:** This case report describes the rare coexistence of cervical cancer with pregnancy, a challenging scenario requiring careful balance between maternal treatment and fetal safety. In Poland, cervical cancer remains a significant health issue, highlighting the need for effective multidisciplinary strategies. **Methods:** This case report was prepared based on CARE guidelines for medical case reporting. The patient was observed by a clinical psycho-oncologist–midwife and a psychologist (also specializing in clinical psycho-oncology) from the start of oncological treatment until delivery and early postpartum. During pregnancy, the pregnant woman was asked three times (at the 23rd, 32nd, and 38th weeks of pregnancy) to complete questionnaires: a self-report questionnaire collecting sociodemographic data, clinical information, and perception of causes and effects of fatigue, the Chalder Fatigue Questionnaire (CHFQ-PL), the Fatigue Management Barriers Questionnaire (FMBQ), the Multidimensional Social Support Scale (MSPSS), and the Walsh Family Resilience Questionnaire (WFRQ-PL). **Results:** The patient, a 37-year-old woman in her second pregnancy, presented with cervical cancer diagnosed in the first trimester. Major concerns included fatigue, emotional distress, and treatment-related uncertainties. Throughout the pregnancy, she underwent four chemotherapy cycles and participated in psycho-oncological assessments to monitor fatigue, which increased as treatment progressed and affected daily functioning and emotional well-being. To enable the early continuation of oncology treatment, the pregnancy was electively terminated by cesarean section at 37+5 weeks, resulting in the good condition of the infant and a stable maternal postpartum condition, though anemia and emotional concerns required further management. **Conclusions:** As research on fatigue in pregnant oncology patients is limited, this case underscores the value of structured psycho-oncological support to enhance care and outcomes for both mother and child.

## 1. Introduction

The coexistence of pregnancy and cervical cancer presents a complex challenge, as it requires managing both maternal and fetal health during cancer treatment. Cancer during pregnancy is rare, affecting about 0.1% of all pregnancies. Cervical cancer poses specific challenges due to its location and the physiological changes induced by pregnancy, which can influence both cancer progression and treatment [1,2]. Over the past decade, treatment strategies have evolved to prioritize pregnancy prolongation and the possibility of delivering a healthy child [3].

In Europe and Poland, cervical cancer remains a significant health issue, with Poland reporting higher rates than many other European countries despite preventive efforts [1,4]. Due to the rarity of cancer during pregnancy, large prospective studies are difficult to conduct, and knowledge is primarily based on retrospective analyses and clinical experience [5].

Pregnant women diagnosed with cervical cancer require multidisciplinary care to balance oncologic treatment efficacy with fetal safety, with treatment options varying by cancer stage and pregnancy trimester [6]. The diagnosis of cancer during pregnancy brings profound emotional and clinical dilemmas for both the patient and her healthcare providers. The need to balance effective cancer treatment for the mother with the safety and well-being of the fetus creates unique challenges. For some, decisions may include to delay treatment, modify it, or, in certain severe cases, consider pregnancy termination to provide the mother with more aggressive treatment options. This decision can be emotionally devastating, particularly for women who deeply desire to continue the pregnancy [7,8].

Fatigue is a well-documented issue for oncology patients, impacting 50–90% and substantially diminishing their quality of life [9]. Despite its prevalence, current guidelines on managing cancer-related fatigue often fail to provide standardized, evidence-based approaches for individualized care, particularly for patients facing multiple challenges, such as those who are pregnant. Additionally, many existing recommendations do not sufficiently address the complex interplay of physical, psychological, and social factors that contribute to fatigue in cancer patients, leading to the under-management of this debilitating symptom. For pregnant cancer patients, fatigue introduces a significant additional burden, impacting daily functioning, well-being, and possibly influencing treatment adherence and outcomes. While fatigue in oncology patients has been widely studied, there is a notable gap in the literature regarding the specific experience of fatigue in pregnant women with cancer. Most existing research focuses on the general oncology population, without considering the compounded impact of pregnancy, which presents unique physical, emotional, and treatment-related challenges. This study addresses this gap by focusing on the intersection of cancer-related fatigue and the physiological changes in pregnancy, a perspective that has not been sufficiently explored in prior studies or case reports. By examining this under-researched issue, the study provides insight into how fatigue management can be tailored to meet the specific needs of pregnant cancer patients. Furthermore, the study emphasizes the importance of psycho-oncological support, which remains a relatively unexplored aspect in the context of fatigue management for this population. The findings of this research offer valuable implications for improving nursing and midwifery practices, providing evidence-based strategies to enhance care outcomes for pregnant oncology patients.

## 2. Materials and Methods

The case report was prepared based on CARE guidelines for medical case reporting [10].

The patient was observed by a clinical psycho-oncologist–midwife and a psychologist (also specializing in clinical psycho-oncology) from the start of oncological treatment until delivery and early postpartum.

At the first meeting, the patient was offered psycho-oncological care and provided with support. The patient consented to participate in the study and prospective follow-up, and to allow access to her medical records. In accordance with the Declaration of Helsinki, approval for the study was obtained from the independent bioethics committee at the Medical University of Gdansk, no. NKBBN/196/2023.

During pregnancy, the pregnant woman was asked three times (at the 23rd, 32nd, and 38th weeks of pregnancy) to complete the following questionnaires:

- A self-report questionnaire collecting sociodemographic data, clinical information, and perception of causes and effects of fatigue.
- The Chalder Fatigue Questionnaire (CHFQ-PL)—a questionnaire adapted to Polish conditions to measure the intensity of fatigue in the physical and mental (cognitive) areas.
- The Fatigue Management Barriers Questionnaire (FMBQ)—a questionnaire to measure patient-perspective barriers to talking about fatigue in the treatment process with healthcare professionals. Permission was obtained from the authors to adapt the tool to Polish conditions.
- The Multidimensional Social Support Scale (MSPSS)—used to measure patients’ perception of the amount and quality of social support they receive from their immediate social environment.
- The Walsh Family Resilience Questionnaire (WFRQ-PL)—a questionnaire adapted to Polish conditions and used to measure family resilience in a difficult situation, which can also be a crisis associated with cancer diagnosis and then continued psycho-oncological and obstetric follow-up until the early postpartum period.

The patient completed the questionnaires independently, in accordance with their intended self-report format.

## 3. Results

### 3.1. Case Summary

#### Patient Information and Medical History

A 37-year-old woman I.B., gravida 2, para 1, with a history of cesarean section in her first pregnancy (5 years previously) due to orthopedic indications (scoliosis), was admitted to the hospital for the management of cervical cancer diagnosed during the current pregnancy. The cancer showed no signs of progression, which allowed for the continuation of the pregnancy without the need for preterm delivery. Her initial pregnancy cytology revealed atypical squamous cells of undetermined significance (ASC-US). The patient has a known allergy to clindamycin and denies any use of stimulants (including nicotine addiction). She reports good living conditions and lives with her family. She has no known family history of genetic diseases. Before the pregnancy, the patient rarely underwent cervical screenings, and approximately six years had passed since her last cytological examination when the disease was diagnosed. The patient had no known risk factors for cervical cancer. The contraceptive method used before pregnancy was the combined oral contraceptive pill.

During the current pregnancy, the patient experienced a range of symptoms, including lower extremity swelling, nausea, vomiting, diarrhea, constipation, and pruritus. In the first trimester of her current pregnancy, she was diagnosed with stage G1 cervical squamous cell carcinoma with microinvasion, accompanied by high-grade squamous intraepithelial lesions (HSILs) in the background. Immunohistochemical staining was positive for p16 and showed high Ki-67 proliferation index (+++/+++). Given the diagnosis, four cycles of chemotherapy were administered at gestational weeks 23, 26, 29, and 32. Throughout the treatment, the patient experienced chemotherapy-related symptoms, including hair loss and fatigue, as well as nausea and vomiting.

Due to a rare genetic defect previously detected in her partner’s child, chorionic villus sampling (CVS) was performed. The results indicated a male karyotype with no evidence of microdeletion or microduplication, confirming no genome imbalance. During the pregnancy, the patient developed anemia and bilateral ovarian cysts, identified through imaging, with characteristics suggestive of dermoid cysts or endometriomas. Additionally, the fetus was diagnosed as being small for gestational age (SGA). In response to this diagnosis, close monitoring was initiated, with ultrasound assessments conducted every three weeks to track fetal growth and well-being. Doppler ultrasound evaluations were also performed to assess blood flow in the umbilical artery and uterine arteries, ensuring optimal placental function. Given the absence of further complications and the stable condition of both the mother and fetus, the pregnancy was concluded as planned at 38 weeks, without the need for preterm delivery. Furthermore, anti-RhE antibodies were detected at a titer of 4 (patient’s Rh status: RhD + Cw−C + c + E−e + K−).

### 3.2. Clinical Timeline

The course of subsequent hospitalizations is shown in Table 1.

Post-delivery observations:

- Delivery outcome: Newborn, 2520 g, 51 cm. Postpartum, mother received analgesics (two doses of intravenous Nalbuphine (20 mg/2 mL) at a 6 h interval and intravenous Paracetamol (1000 mg/100 mL) four times a day at 6 h intervals, which provided noticeable pain relief); lactation was inhibited per her request (non-pharmacological methods, but on the fourth postpartum day, the onset of milk let-down was noticed, and pharmacological inhibition of lactation with Cabergoline (0.5 mg) was initiated). The newborn was fed with milk from a breast milk bank through a bottle with a pacifier.
- Psycho-oncological findings: Observed lower mood; concerns about treatment. The patient scored 13 on the Edinburgh Postpartum Depression Scale, indicating increased risk.
- Discharge: Due to severe leg edema, the patient was advised to rest with her legs elevated. As part of anticoagulant prophylaxis, the patient received daily subcutaneous Enoxaparin (40 mg/0.4 mL). From the first day of postpartum, the patient showed signs of anemia, and her hemoglobin levels were monitored daily with the following results: 8.8 g/dL, 8.5 g/dL, and 8.3 g/dL. Pharmacological treatment with Ferrous Sulfate (80 mg twice daily) was introduced. Due to the need for phototherapy in the newborn and the mother’s anemia, the obstetric patient and her baby were discharged on the seventh day following the cesarean section. Follow-up in 4–6 weeks was advised for oncological treatment.

### 3.3. Psycho-Oncological Evaluation

A summary of the psycho-oncological evaluation is presented in Table 2.

### 3.4. Patient Perspective

The patient expressed satisfaction with the support received from the midwife and described her home environment as conducive to recovery.

## 4. Discussion

This case report highlights the complexity of managing cervical cancer during pregnancy, particularly when balancing maternal treatment with fetal safety in nursing and midwifery practice. Psycho-oncological support played a significant role in this patient’s care, particularly given her elevated risk for postpartum depression and anxiety related to future oncological treatment. A key strength of this approach was the integration of psycho-oncological assessments that tracked fatigue and emotional well-being prospectively. This allowed for a detailed understanding of how fatigue sources evolved, shifting from stress related to the diagnosis to physical exhaustion due to chemotherapy, which aligns with existing findings on cancer-related fatigue in non-pregnant oncology patients [11,12].

In midwifery practice, it is essential to support both mother and child throughout pregnancy and the postpartum period. Women facing cancer during pregnancy or within a year postpartum experience two transformative and paradoxical life events simultaneously: bringing new life into the world while confronting their own mortality. The diagnosis and treatment of cancer in pregnancy present unique challenges alongside those of pregnancy, childbirth, and parenting [13]. Pregnant oncology patients and their families often face conflicting emotions, as they must prioritize either the mother’s or the child’s life by making complex decisions regarding imaging methods, fetotoxicity risks of chemotherapy, adverse maternal outcomes, and the risk of preterm delivery as a treatment side effect [14]. Fatigue significantly affects health-related quality of life, as cancer patients may be too exhausted to engage fully in daily activities and to fulfill previously held roles [15].

This patient was asked to subjectively assess her own health on a scale from 0 to 10, where 0 represents very poor health and 10 excellent health. During the initial assessment, she rated her health as 8. This rating appears unusually high given the medical data regarding her condition. A 2021 national health report indicated that over 66% of individuals rated their health as good or very good, with the most commonly reported ailments being lower back pain and high blood pressure [16].

Such a high health rating by our patient may be associated with various phenomena, ranging from defense mechanisms that distort reality (making it appear less threatening) to the protective influence of hope linked to optimistic prognoses, despite a serious illness coexisting with pregnancy [17].

Throughout the support process, the patient’s subjective health rating gradually declined from 7 to 6. She believed that both her close contacts and medical staff shared this perception. Her health-related anxiety remained high, initially at 8, briefly decreasing to 5, before returning to 8 in the third assessment. The results indicate a progressive increase in fatigue severity over time, as reflected in the assessments. Studies on oncological patient groups using the same tool indicate that patients not reporting fatigue as a significant issue typically scored around 17 (group median), while those for whom fatigue was a major concern scored approximately 27 points on the questionnaire [18]. In both the cited studies and our case, the patients’ responses on this questionnaire were coded using Likert scoring, with 33 representing the maximum score (indicating the highest level of fatigue) achievable on this questionnaire.

At each assessment, our patient consistently perceived that her own fatigue ratings were closely aligned with those of her family and healthcare providers, with no significant differences among these perspectives. She discussed her fatigue primarily—and almost exclusively—with her family throughout each stage of the study.

To better understand communication processes related to fatigue, the FMBQ was utilized to measure barriers to discussing fatigue with medical personnel. The assessment of communication barriers revealed specific difficulties in expressing fatigue-related concerns. These findings suggest that the presence of these barriers may not be solely attributed to patient personality factors but may also relate to current healthcare experiences. Ultimately, we observed a notable increase in barriers preventing our patient from bringing up fatigue during interactions with medical staff.

In unpublished research involving a Polish adaptation of this questionnaire, the mean score among a similar cohort of women hospitalized for gynecological cancer was M = 79.93 (SD = 15.7), suggesting that our patient’s barrier intensity does not differ significantly from the typical oncology patient. The minimum possible score on this questionnaire is 28 (indicating a complete absence of barriers to discussing fatigue with medical personnel), while the maximum score is 140 (indicating the highest possible barrier intensity). Although there are no established norms for this questionnaire, our patient’s score falls within the range of average barrier intensity.

Throughout treatment, certain barriers remained stable, particularly the patient’s reluctance to discuss fatigue due to fears related to disease progression, mental health stigma, and potential treatment disruption. Among these consistently maintained barriers, the strongest was her preference for non-pharmacological approaches to managing fatigue (even if these originate from alternative medicine).

Minor fluctuations were observed in her desire to be a “good patient” who does not complain and her fear of distracting the physician, who she believes should primarily focus on treating her cancer rather than ancillary concerns.

Significant changes occurred in the remaining subscales. Notably, the patient’s sense of futility regarding treatment and the discussion of fatigue—believing that nothing can be done about it—increased. Similarly, general medical concerns about fatigue treatment, such as fears of additional medications or potential interactions with her ongoing cancer treatment (which she wished to minimize), intensified.

A substantial increase was observed in the subscale for lack of communication with medical personnel regarding fatigue, effectively doubling. This result suggests a growing perception in the patient that medical personnel showed little interest in her fatigue concerns; they certainly did not initiate discussions on this matter, and there were no direct inquiries about it in daily communication—a situation that evolved over the course of her treatment. The patient began treatment with a sense of contact on this issue but ended with a strong conviction that this connection was lacking.

The only subscale where a decrease was observed pertained to her belief that fatigue was not a reason for concern, given that there were more pressing issues, such as her illness and its treatment.

Fatigue was generally not a factor that hindered treatment for the patient, though her perception of it showed some variation. In the first assessment, she rated fatigue as having no impact, while in the second and third assessments, she rated it as impactful. However, her perception of the primary cause of her fatigue changed throughout treatment. Initially, she attributed her fatigue primarily to the stress of her illness and treatment. In the second assessment, she perceived her fatigue as mainly due to physical weakness from the illness itself, viewing it as a natural response of her body to being ill. By the third assessment, she identified the treatment process itself as the most exhausting factor, citing the series of logistical demands (attending appointments and undergoing medical procedures at various locations) that significantly disrupted her family’s usual daily schedule.

Despite this, the patient reported that her fatigue generally did not interfere with daily functioning or meeting her family’s needs, rating its impact as low (2 in the first and 3 in the third assessments). The exception was the second assessment, where she perceived a stronger impact of fatigue on daily life and her ability to meet family needs.

Given the severe, maximum level of fatigue and its minimal impact on the treatment process or daily functioning, compensatory and protective mechanisms must be in place for this patient. Social support could be one such mechanism. The patient reported substantial support from close relations in areas involving understanding. According to her, her loved ones had a strong grasp of her fatigue and offered tangible assistance. This level of support remained relatively stable throughout the treatment period.

The patient reported a strong perception of social support, which may have influenced her coping strategies. This suggests a very high, effectively maximal, sense of social support across all dimensions—support from family, friends, and other significant individuals in the patient’s life.

Confirmation of these findings is essentially provided by the final questionnaire used in our case study, the WFRQ-PL, which highlights the resilience emerging in the patient’s family during this crisis. The family’s belief system was identified as a key resilience factor, with family members demonstrating a strong ability to interpret challenging situations positively and collectively. This allowed them to approach adversity as a shared challenge, fostering mutual support and collaboration. The belief system relates to how the family as a whole perceives and interprets challenging situations and crises. In the face of adversity, the patient’s family seemed to employ strategies such as attributing meaning to hardships, maintaining a positive outlook, and drawing on transcendence and spirituality. This approach fosters a perception of the crisis as a shared challenge, one that can be overcome through mutual engagement, collaboration, and normalization of each family member’s responses. The family members allow themselves to experience difficult emotions and support one another, creating a sense of unity and harmony with the world and nature.

The family displayed strong organizational processes, including flexibility, adaptability, and leadership. They were able to maintain stability and continuity despite the challenges posed by the patient’s condition.

In the communication processes subscale, the family consistently scored 40 out of a possible 50 points across all three assessments. This subscale provides insight into the clarity and transparency of the family’s communication (coherent, unambiguous, and direct expression of needs), the openness of emotional expression (sharing experienced emotions, tolerating the feelings of others, and spending time together), and their collaborative problem-solving (joint planning, exploring options, and supporting one another’s decisions and actions).

It appears that these resilience resources within the patient’s family, further mobilized in response to the illness and treatment, were integral to her ability to manage such a high level of fatigue effectively.

Concerns regarding the future and dilemmas about carrying the pregnancy to term added to this complexity. Our patient expressed feelings of futility about the treatment, and our assessments indicated that her rising levels of fatigue began to interfere with her ability to undergo treatment. The midwife’s role is to ensure the safety of both mother and child. According to the Organizational Standard for Perinatal Care [19], midwives are required to monitor the mental health of pregnant and postpartum patients. Routine postpartum depression screening is now standard practice; however, clinically significant factors that may heighten the risk of depression are often overlooked. Fatigue in cancer patients, though well studied, is still not sufficiently addressed in pregnant oncology patients. In our study, we observed that the patient reported high levels of anxiety and increased fatigue over time. Previous research on fatigue in gynecologic oncology patients has indicated that younger patients—those often of reproductive age, like our case patient—report fatigue more frequently than older patients. Additionally, those experiencing fatigue often report higher levels of anxiety and depression. Fatigue in this demographic is strongly associated with diminished quality of life, as measured across multiple domains of the SF-36 scale, highlighting the specific vulnerabilities of younger patients managing both oncology treatment and pregnancy-related demands. Research on the role of midwives in supporting pregnant cancer patients underscores their crucial role in treatment decision-making and continuity of care through to neonatal care [14,20]. In the context of our study, this aspect is particularly important, as we observed shifts in the patient’s sources of fatigue—from stress related to the diagnosis to physical weakness due to treatment. Addressing this element of care continuity is crucial for effective care planning.

This case illustrates that a well-coordinated, multidisciplinary care model can support positive outcomes for both mother and child in cases of cervical cancer during pregnancy. Structured psycho-oncological support proved beneficial, addressing psychological symptoms and providing a framework for managing the compounded effects of cancer and pregnancy on quality of life. This model underscores the necessity of nursing and midwifery involvement in managing fatigue and emotional health, as well as the importance of family support as part of a comprehensive treatment plan. A growing body of research highlights the effectiveness of various nursing interventions (especially with the support of a multidisciplinary team) in combating fatigue, which is a prevalent issue for cancer patients. Nurses and midwives, with their holistic approach to patient care, are uniquely positioned to address not only the physical but also the psychological aspects of fatigue. Studies have demonstrated that interventions such as tailored physical activity programs, psychoeducation, and emotional support can significantly reduce fatigue and improve quality of life in oncology patients [21,22,23,24,25]. Furthermore, nursing care emphasizes the importance of patient-centered approaches, which can facilitate ongoing support throughout treatment, helping patients manage fatigue more effectively. As fatigue is often a complex, multifactorial symptom, the inclusion of nursing and midwifery professionals in the care team is crucial to providing comprehensive, continuous care, ensuring that patients’ physical, emotional, and social needs are adequately met.

It appears that even broader perspectives are opened through the collaboration between nurses and the patient’s family. Research on the collaboration between nurses and families, particularly in the context of cancer patients, confirms that this approach is especially effective in managing fatigue. Involving the family in the care process and working together with the nurse or midwife allows for a more personalized approach, increasing both emotional and physical support for patients. Joint efforts in health education, symptom monitoring, and encouraging adherence to rehabilitation plans significantly improve patients’ quality of life and the effectiveness of managing fatigue associated with cancer and its treatment. Family collaboration significantly reduces cancer-related fatigue, improving treatment outcomes and patients’ functioning in daily life [26]. A strength of this case report is the holistic and individualized care plan, which included psycho-oncological support alongside physical cancer management. The applied assessment tools provided valuable insights into the psychological aspects influencing the patient’s experience of fatigue. Clinical decision-making in the management of cervical cancer during pregnancy is guided by established protocols, which consider various factors, including the patient’s tumor stage, gestational age, and personal preferences regarding pregnancy preservation. Recommendations emphasize a multidisciplinary approach, with treatment options tailored to the individual case. For early-stage cancers (such as stage IA1), pregnancy preservation may be possible, with treatment deferred until postpartum. In cases of more advanced disease, neoadjuvant chemotherapy, surgery, or pregnancy termination may be considered based on tumor size and progression. The timing of chemotherapy or surgery depends on the tumor stage and fetal viability, with a focus on minimizing fetal risk. These decisions are underpinned by a careful assessment of diagnostic tests, including cytological, colposcopic, and imaging evaluations, and must prioritize both maternal and fetal health. Therefore, the clinical management of cervical cancer during pregnancy requires a personalized and evidence-based approach, balancing the risks and benefits of treatment options at each stage of pregnancy [27].

Incorporating psycho-oncological support into the management of pregnant women with cervical cancer is essential due to the profound psychological challenges posed by this diagnosis. Pregnancy and cancer intersect to create a unique emotional burden, with patients experiencing heightened anxiety, stress, and uncertainty. These factors can significantly impact their decision-making, emotional well-being, and adherence to treatment. Psycho-oncological support provides a vital space for patients to process their emotions, develop coping mechanisms, and receive guidance on navigating the complex treatment landscape. Furthermore, psychological care has been shown to improve overall health outcomes by reducing stress levels, which can positively influence treatment responses [13,15,18]. Integrating psycho-oncological care into the multidisciplinary approach not only addresses the patients’ physical health needs but also ensures their mental and emotional needs are met, supporting their resilience throughout the treatment process.

In nursing and midwifery practice, special attention should be given to psycho-oncological support, which should include a regular assessment of the emotional state of patients, especially in the context of fatigue related to oncological treatment and pregnancy. Nurses and midwives should also support patients in making difficult decisions about treatment and pregnancy continuation, taking into account their preferences and needs. Training nursing staff to recognize and respond to the emotional needs of women with cancer during pregnancy is crucial for providing holistic care that addresses both the physical and emotional needs of these patients.

However, certain limitations must be acknowledged. While the comprehensive care model yielded positive maternal and fetal outcomes, the extensive resources involved—namely, psycho-oncological support and frequent evaluations—may limit the generalizability of this approach in resource-constrained settings. Additionally, given the rarity of cervical cancer in pregnancy, this case cannot provide the breadth of insight that larger cohorts might offer, leaving certain findings open to interpretation. A long-term follow-up for both mother and child would further strengthen the conclusions, particularly regarding potential developmental outcomes for the child after in utero exposure to chemotherapy.

## 5. Conclusions

The lessons learned from this case emphasize the importance of prospective fatigue monitoring and the integration of psycho-oncological support. Our findings, particularly our patient’s evolving fatigue levels as reflected in the psycho-oncological assessments (CHFQ-PL, FMBQ, and WFRQ-PL), highlight the need for ongoing emotional and psychological support. The patient’s physical and emotional burdens changed over time, with fatigue intensifying as treatment progressed, which further underscores the importance of regular monitoring and tailored psychological interventions. This case supports the notion that managing both physical symptoms and psychosocial needs is essential to providing quality care.

## Figures and Tables

**Table 1:** The course of subsequent hospitalizations.

Admission	Gestational Week	ICD-10 Diagnosis	Assessment and Observations	Interventions
First Admission	23 weeks	O26.9—Pregnancy with cervical cancer; O34.2—Maternal care post-cesarean; O36.5—Fetal growth concern; O99.0—Anemia complicating pregnancy	General condition good, fully oriented, well nourished, pink skin, lower extremity swelling, no bleeding or amniotic fluid leakage.	Initiated psycho-oncological support. Patient agreed to regular fatigue and emotional assessments. Treatment: Dexamethasone phosphate 18 mg, Paclitaxel 145 mg, Carboplatynum 625 mg, Pegfilgastrim 6 mg, Clemastin 1 mg.
Follow-Up	26 weeks	As above	General condition stable, continued lower extremity swelling, FIGO category I on cardiotocography (CTG).	Continued psycho-oncological support. Treatment: Dexamethasone phosphate 18 mg, Paclitaxel 145 mg, Carboplatynum 625 mg, Pegfilgastrim 6 mg, Clemastin 1 mg.
Follow-Up	29 weeks	As above	Condition stable, lower extremity swelling, no fluid leakage or bleeding, FIGO category I on CTG.	Ongoing psycho-oncological support and assessment. Treatment: Dexamethasone phosphate 18 mg, Paclitaxel 145 mg, Carboplatynum 625 mg, Pegfilgastrim 6 mg, Clemastin 1 mg.
Follow-Up	32 weeks	As above	General condition stable, observations as previous. FIGO category I on CTG.	Continued psycho-oncological support and assessment. Treatment: Dexamethasone phosphate 18 mg, Paclitaxel 145 mg, Carboplatynum 625 mg, Pegfilgastrim 6 mg, Clemastin 1 mg.
Final Admission	38 weeks	O26.9—Pregnancy with cervical cancer; O34.2—Maternal care post-cesarean; O36.5—Fetal growth concern; O99.0—Anemia complicating pregnancy; O82.0: Single birth by elective cesarean section.	Comprehensive assessment: single live fetus in cephalic position, estimated fetal weight in the 9th percentile. Hemoglobin 10.9 g/dL. Planned cesarean section for oncological treatment post-delivery.	Continued psycho-oncological support and assessment. Cesarean delivery conducted (transperitoneal suprapubic transverse cesarean section via laparotomy using the Misgav Ladach technique). Newborn: 2520 g, 51 cm. Postpartum psycho-oncological support. Treatment: Enoxaparin injection 40 mg/0.4 mL, Ferrous sulfate tablets 80 mg iron, Electrolyte solution for infusion, Cabergoline tablets 0.5 mg, Cefazolin injection 1000 mg, Ibuprofen capsules 200 mg, Ketoprofen injection 100 mg/2 mL, Nalbuphine injection 20 mg/2 mL, Paracetamol injection 1000 mg/100 mL

**Table 2:** Psycho-oncological evaluation.

	Measurement 1 (18 August 2023)	Measurement 2 (22 October 2023)	Measurement 3 (28 November 2023)	Commentary/ Interpretation
Health status	8	7	6	Health is deteriorating
Patient–family health assessment concordance	Rather yes	Yes	Rather yes	
Patient–clinician health assessment concordance	Rather yes	Yes	Rather yes	
Anxiety level related to health situation	8	5	8	Anxiety level very high
CHFQ-PL	30	32	33	Fatigue increases slightly
Patient–family fatigue assessment concordance	Rather yes	Yes	Rather yes	
Patient–clinician fatigue assessment concordance	Rather yes	Yes	Rather yes	
Main source of fatigue	Stress related to illness and treatment	Disease-related weakness (fatigue is part of the disease)	Treatment of the disease (organizing)	The source of fatigue is changing
Fatigue makes treatment more difficult now	0	3	3	Fatigue begins to hinder treatment
Fatigue makes daily life difficult	2	5	3	Fatigue increases slightly, making daily life more difficult
Fatigue makes it difficult to meet family needs	2	5	3	Similarly, meeting the needs of the family
Relatives understand fatigue and support	9	8	8	Relatives understand, but less
With whom patient talks about fatigue	With family	With family	With family	Patient talks to family
FMBQ	74	66	80	Barriers are growing
Meaningless treatment	10	8	12	Growing
Fear of progression	4	4	4	
Being a good patient	12	11	12	
Fear of distracting the doctor	6	4	6	
No worries	14	10	12	Decreasing
Fear of the stigma	4	4	4	
General medical concerns	8	9	10	Growing
Preference for non-medical interventions	8	8	8	
Fear of interfering with treatment	4	4	4	
Lack of communication	4	4	8	Growing strongly
Total support	7	7	7	
Support from Friends	7	7	7	
Support from Family	7	7	7	
Support from Significant Person	7	7	7	
WFQ-R beliefs	52/65	52/65	60/65	
WFQ-R organizational processes	30/40	30/40	34/40	
WFQ-R communication processes	40/50	40/50	40/50	

## Data Availability

The original contributions presented in the study are included in the article, and further inquiries can be directed to the corresponding author.

## Public Involvement Statement

There was no public involvement in any aspect of this research.

## Guidelines and Standards Statement

The case report was prepared based on CARE guidelines for medical case reporting [10].

## Abbreviations

The following abbreviations are used in this manuscript:

ASC-US	Atypical squamous cells of undetermined significance
CHFQ-PL	Chalder Fatigue Questionnaire
FMBQ	Fatigue Management Barriers Questionnaire
MSPSS	Multidimensional Social Support Scale
WFRQ-PL	Walsh Family Resilience Questionnaire
